# Cognitive Decline in Older Persons Initiating Anticholinergic Medications

**DOI:** 10.1371/journal.pone.0064111

**Published:** 2013-05-31

**Authors:** Raj C. Shah, Alicia L. Janos, Julia E. Kline, Lei Yu, Sue E. Leurgans, Robert S. Wilson, Peter Wei, David A. Bennett, Kenneth M. Heilman, Jack W. Tsao

**Affiliations:** 1 Rush Alzheimer's Disease Center (RADC), Rush University Medical Center, Chicago, Illinois, United States of America; 2 Department of Family Medicine, Rush University Medical Center, Chicago, Illinois, United States of America; 3 Department of Neurology, Uniformed Services University of the Health Sciences, Bethesda, Maryland, United States of America; 4 Department of Neurological Sciences, Rush University Medical Center, Chicago, Illinois, United States of America; 5 Department of Behavioral Sciences, Rush University Medical Center, Chicago, Illinois, United States of America; 6 Department of Neurology, University of Florida and Malcom Randall Veterans Affairs Medical Center, Gainesville, Florida, United States of America; 7 United States Navy Bureau of Medicine and Surgery, Washington, DC, United States of America; “Mario Negri” Institute for Pharmacological Research, Italy

## Abstract

**Background:**

This study examines the effect of initiating medications with anticholinergic activity on the cognitive functions of older persons.

**Methods:**

Participants were 896 older community-dwelling, Catholic clergy without baseline dementia. Medication data was collected annually. The Anticholinergic Cognitive Burden Scale was utilized to identify use of a medication with probable or definite anticholinergic activity. Participants had at least two annual cognitive evaluations.

**Results:**

Over a mean follow-up of 10 years, the annual rate of global cognitive function decline for never users, prevalent users, and incident users was −0.062 (SE = 0.005), −0.081(SE = 0.011), and −0.096 (SE = 0.007) z-score units/year, respectively. Compared to never users, incident users had a more rapid decline (difference = −0.034 z-score units/year, SE = 0.008, p<0.001) while prevalent users did not have a significantly more rapid decline (p = 0.1).

**Conclusions:**

Older persons initiating a medication with anticholinergic activity have a steeper annual decline in cognitive functioning than those who are not taking these medications.

## Introduction

Cognitive decline frequently occurs in older persons [1] and is associated with adverse health consequences, including increased disability, dependence, and death [2]. With the increase in the percent of older Americans, it is important to identify modifiable risk factors related to cognitive decline.

Acetylcholine plays a critical role in the synaptic plasticity necessary for learning and memory. Medications with anticholinergic activity commonly are used by older persons and have been associated with cognitive impairment in cross-sectional studies [3]–[6]. While limited [7], most [8]–[10], but not all [11], of the reported longitudinal studies in community-dwelling elders have shown prevalent anticholinergic use to be associated with cognitive decline. However, such study designs are limited in that they are not able to determine if initiation of a medication with anticholinergic activity results in a change in the trajectory of cognitive decline in older persons.

In order to determine whether initiating the use of a medication with anticholinergic activity is associated with more rapid cognitive decline, we used data from 896 community-dwelling older clergy without dementia who were participating in the Religious Orders Study [12], a longitudinal epidemiologic study of aging where participants have been assessed annually for a mean of ten years. At baseline and during annual evaluations, medication use was recorded, and each participant underwent a detailed assessment of cognitive functions.

## Methods

### Ethics Statement

The Religious Orders Study was approved by the Rush University Medical Center Institutional Review Board. Written informed consent was obtained from all study participants.

### Participants

Participants were older Catholic nuns, priests or lay brothers from approximately 40 groups across the United States [12]. From January 1994 to March 2012, 1096 participants completed baseline and annual follow-up evaluations which included a medical history, neurological examination, assessment of cognitive functions, and clinical classification of dementia and Mild Cognitive Impairment (MCI), as previously described [13], [14]. Dementia required a history of cognitive decline and impairment in at least two cognitive domains [15]. Impairment in at least one cognitive domain in the absence of dementia was classified as MCI [16]. Baseline dementia was present in 83 individuals, baseline stroke or Parkinson's disease was present in 76 individuals, 23 died before the first follow-up evaluation, and 18 had only the baseline visit at time of analysis. After excluding these participants, the remaining 896 persons completed a mean of ten annual evaluations (maximum follow-up 18 years). Cognitive functions were assessed in almost 95% of all possible follow-up evaluations.

### Assessment of cognitive functions

At each annual evaluation, a battery of 19 cognitive function tests was administered in addition to the Mini-Mental State Examination (MMSE) [17]. The battery included seven tests for episodic memory: Word List Memory, Word List Recall, and Word List Recognition; immediate and delayed recall of Story A from Logical Memory; and, immediate and delayed recall of the East Boston Story. The other twelve tests were: Alpha Span, a 20-item Boston Naming Test, Digit Span Forward, Digit Span Backward, Digit Ordering, a 15-item version of Extended Range Vocabulary, a 15-item form of Judgment of Line Orientation, a 20-item form of the National Adult Reading Test, Number Comparison, a 17-item form of Standard Progressive Matrices, the oral version of the Symbol Digit Modalities Test, and Verbal Fluency [18]. Raw scores from the 19 individual tests were converted to z-scores, using the mean and standard deviation from the baseline evaluation of all participants. We constructed summary measures of global cognitive function by averaging the z-scores of all 19 individual tests, as previously described [18]. We constructed a summary measure of episodic memory by averaging the z-scores from the seven episodic memory tests.

### Use of medications with anticholinergic activity

At each evaluation, study personnel recorded all medications taken by the participants during at least the previous two weeks. A master list of medications defined as having probable or definite anticholinergic activity was constructed using the Anticholinergic Cognitive Burden Scale [9]. We first divided our participants into two groups (prevalent users and non-prevalent users) and then divided the non-prevalent users into two groups (never users and incident users). The participants classified as never users did not report the use of any medications with anticholinergic activity during the study period. Those in the prevalent user category were using at least one medication with anticholinergic activity at the time of enrollment. Incident users were defined as those who first reported using a medication with anticholinergic activity after enrollment.

### Other covariates

Date of birth, sex, and years of education completed were recorded for each participant. Apolipoprotein E (ApoE) genotyping was done using methods adapted from Hixson and Vernier [19], and we classified participants as either having or not having at least one ε4 allele. To examine the burden of medical illness, the presence of the seven most common chronic medical conditions in the cohort was determined by self-report (cancer, head injury, thyroid disease) or by clinician review of (myocardial infarction, diabetes, hypertension, stroke) [14]. Urinary incontinence was determined by self-reported urine loss throughout the previous month. Depressive symptoms experienced during the previous week were assessed using a 10-item version of the Center for Epidemiologic Studies Depression Scale [20]. Physical activity was based on self-report regarding the number of hours of activity during the past two weeks [14], [21]. Needing assistance with activities of daily living (ADL) was assessed using a modified version of the Katz Index of Independence in ADL [22], [23].

### Statistical analysis

Analyses were carried out in SAS®, Version 9.3 (SAS Institute Inc., Cary, NC). We compared baseline demographic and covariate measures of the three groups (never users, prevalent users, and incident users) using an analysis of variance (ANOVA).

We used mixed-effects models [24] adjusted for age, gender, and education level to compare the trajectory of cognitive function change for prevalent users as compared to non-prevalent users. Next, we split the non-prevalent users into never users and incident users and compared the rate of cognitive function change among prevalent users, incident users, and never users. In an exploratory analysis, we allowed the rate of change in global cognitive functions to differ before and after initiation of a medication with anticholinergic activity (see Tables S1, S2 and S3 for an explicit description of the model specification). We then compared the annual rate of change in cognitive function post-initiation of a medication with anticholinergic activity with the annual rate of cognitive change in (1) incident users before initiation of a medication with anticholinergic activity, (2) prevalent users, and (3) never users. We repeated the models by replacing global cognitive functions with episodic memory function.

In secondary analyses, we repeated the mixed effect model focusing on incident users only. We examined whether the difference in the annual rate of change in global cognitive functions pre- and post-use of a medication with anticholinergic activity was affected by baseline covariates, including presence of an ApoE ε4 allele, MCI, number of chronic medical conditions, urinary incontinence, number of depressive symptoms, physical activity, and disability on the Katz Index.

## Results

### Baseline characteristics of cohort

There were 569 never users, 90 prevalent users, and 237 incident users; see Table 1 for summaries of baseline characteristics. The majority (88%) of prevalent users took a single medication with anticholinergic activity; 12% took two such medications. Use of meclizine, paroxetine, tolterodine, amitriptyline, or carbamazepine categorized 58% of the participants in the prevalent users group. After baseline, 21 participants (23%) in the prevalent users group no longer took a medication with anticholinergic properties. Compared to the never user group, the prevalent users were older, were more likely to be women, had lower MMSE scores, reported urinary incontinence and more depressive symptoms, were less physically active, and had more disability on the Katz Index.

**Table 1:** Baseline Characteristics of the Participants, According to Study Group.*

Variable	Prevalent Users (n = 90)	Non-Prevalent Users (n = 806)		P-value and test statistic
		Incident Users (n = 237)	Never Users (n = 569)	
Age – yr	76.5±7.5	75.9±6.5	74.0±7.0	p<0.001, F_2;892_ = 9.55
Female – no. (%)	72 (80.9)	178 (75.1)	371 (65.3)	p = 0.001, X^2^_2_ = 13.6
Education – yr	17.9±3.0	18.1±3.4	18.2±3.6	p = 0.7, F _2;894_ = 0.3
Mini-Mental State Examination – score†	28.1±1.9	28.4±1.7	28.7±1.6	p = 0.004, X^2^_2_ = 11.0
Presence of ApoE ε4 – no./total no. (%)	15/77 (19.5)	70/216 (28.1)	107/477 (22.4)	p = 0.010, X^2^_2_ = 9.3
Mild Cognitive Impairment – no. (%)	23 (25.6)	67 (28.2)	130 (22.9)	p = 0.3, X^2^_2_ = 2.7
Conversion from Mild Cognitive Impairment to dementia during study period – no. (%)	14 (60.9)	38 (56.7)	63 (48.5)	p = 0.4, X^2^_2_ = 2.0
Number of chronic medical conditions reported, out of 7‡	1.1±1.0	1.1±1.0	1.0±1.0	p = 0.2, X^2^_2_ = 2.8
Urinary Incontinence – no. (%)	49 (55.1)	133 (56.3)	233 (41.0)	p<0.001, X^2^_2_ = 18.7
CES-D Score§	1.5±2.0	1.3±1.6	0.8±1.3	p = <0.001, X^2^_2_ = 25.7
Physical Activity – hours/week	1.7±1.9	3.2±5.0	3.0±3.8	p = 0.006, X^2^_2_ = 10.2
Katz Index of Independence in Activities of Daily Living&	0.3±0.8	0.1±0.4	0.0±0.3	p<0.001, X^2^_2_ = 32.2

* Plus-minus values are means ± SD. ApoE denotes apolipoprotein E genotype.

† The Mini-Mental State Examination score has a maximum value of 30, with higher scores indicating better performance.

‡ Chronic medical conditions considered are history of cancer, diabetes, head trauma, hypertension, myocardial infarction, stroke, and thyroid disease.

§ CES-D is the score on the Center for Epidemiologic Studies Depression Scale. Range is 0 to 10 with higher scores indicating more depressive symptoms.

& Katz Index of Independence in Activities of Daily Living is number of six activities (feeding, bathing, dressing, toileting, transferring, and ambulating) reported as needing help or unable to do.

The majority (95%) of incident users initially took a single medication with anticholinergic activity, with 5% taking two medications. Use of oxybutynin, meclizine, tolterodine, paroxetine, or amitriptyline categorized 61% of the participants in the incident users group. After initiating a medication with anticholinergic activity, 75 of the 215 participants with follow-up data (36%) in the incident user group no longer reported taking a medication with anticholinergic properties in their subsequent annual evaluations. Initiation of a medication with anticholinergic activity occurred a mean of 4.5 years after study enrollment (SD = 2.9). Compared to never users, incident users were older, more likely to be women, report urinary incontinence and more depressive symptoms, and more likely to have an ApoE ε4 allele.

### Medications with anticholinergic activity and change in global cognitive functions

**Figure 1:**
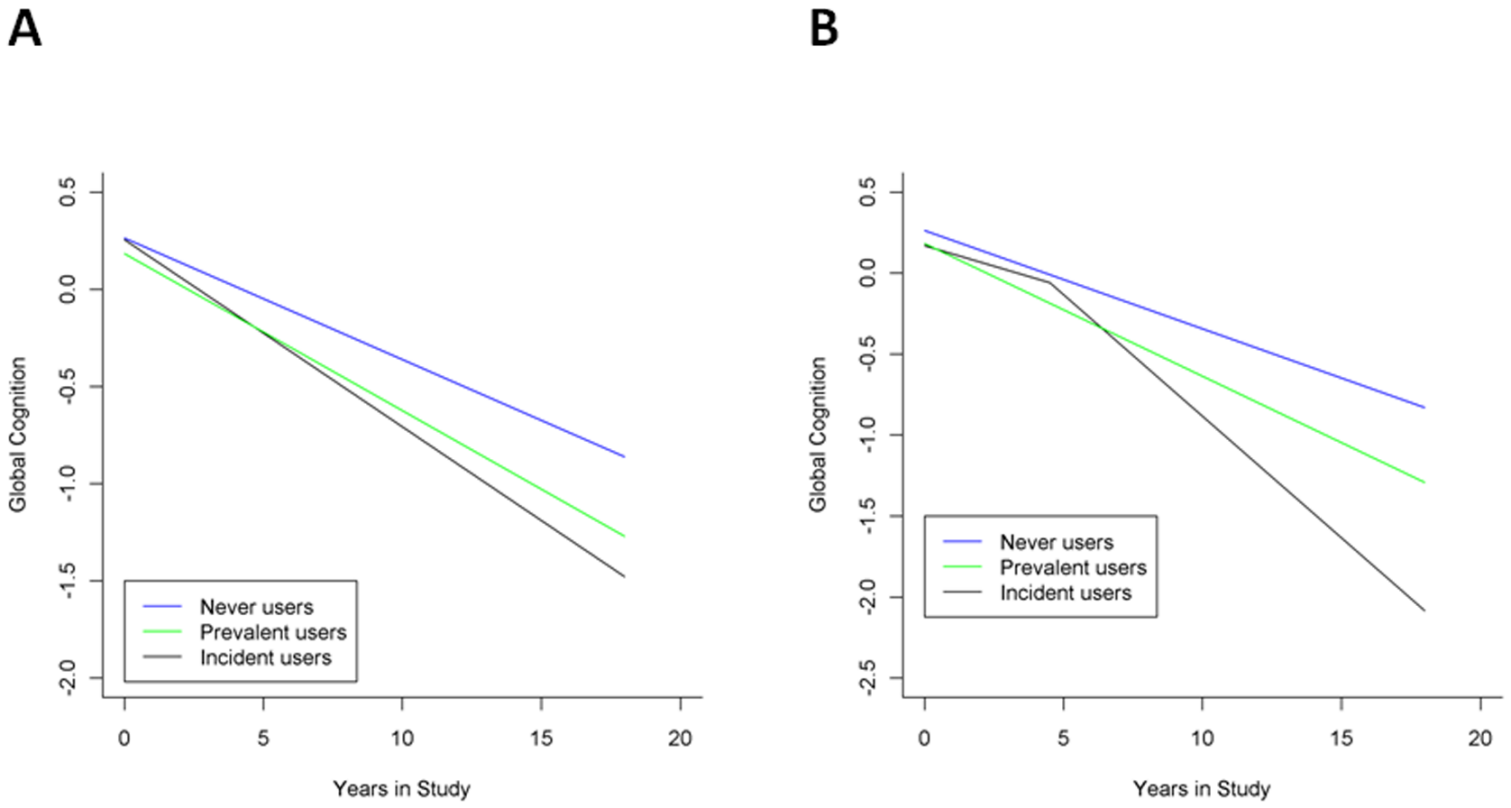
Estimated global cognitive performance (z-score units) as a function of time in study. Figure 1A shows results from a mixed-effects model without a change point added for initiation of a medication with anticholinergic activity and Figure 1B shows results from a model with a change point. The blue line shows average global cognitive performance over time for a typical person (75-year-old female with 18 years of education) who never used an anticholinergic medication. The green line shows average cognitive function over time for a typical person who was taking a medication with anticholinergic activity at baseline. The black line shows average global cognitive function over time for a typical person who first started anticholinergic medication after 4.5 years.

As prior studies in community-dwelling older persons have focused on comparing the cognitive trajectories in prevalent users to non-prevalent users [8]–[11], we first compared the annual rate of change in global cognitive functions using a mixed-effects model adjusted for age, gender, and education level and found no significant difference (parameter estimate for difference = −0.007 z-score units/year, SE = 0.012, p = 0.6). As non-prevalent users represent a combined group of incident users and never users, we repeated the model to compare the rate of change in cognition for the three groups and plotted the trajectory for the average participant in each group (see Figure 1A). As listed in Table 2, the annual rate of change in global cognitive functions showed a gradient with greatest decline for incident users and the least decline for never users. Compared to never users, incident users did have a more rapid decline (difference = −0.034 z-score units/year, SE = 0.008, t_7420_ = −4.18, p<0.001); however, prevalent users did not differ in annual rate of change in global cognitive functions (difference = −0. 018 z-score units/year, SE = 0.012, t_7420_ = −1.47, p = 0.1). Finally, as the incident user annual rate of change in global cognitive functions is an amalgam of pre-use and post-use rates of change, we conducted an exploratory analysis by allowing different rates of cognitive change before and after first use of a medication with anticholinergic activity (see Figure 1B). The annual rate of change in global cognitive functions shows a gradient with the greatest decline for incident users was after initiation and the least annual rate of decline for incident users was before initiation (see Table 2). No significant difference was found between the pre-use slope of incident users and that of never users (p = 0.2) and for prevalent users as compared to never users (p = 0.2). Post-use annual rate of cognitive change in incident users was 1.8-fold more rapid than never users (difference = −0.089 z-score units/year, SE = 0.013, t_207_ = −7.06, p<0.001). For incident users, the post-use annual rate of cognitive change was 2.9-fold more rapid than prior to starting medication with anticholinergic activity (difference = −0.099 z-score units/year, SE = 0.012, t_207_ = −8.35, p<0.001).

**Table 2:** Annual rate of change in global cognitive functions by user group.

	Annual Rate of Change in Global Cognitive Functions (SE), z-score units/year		
Model:*	A	B	C
**User Type:**			
Prevalent User	−0.081 (0.012)	−0.081 (0.011)	−0.082 (0.017)
Non-Prevalent User	−0.074 (0.004)		
Incident User		−0.096 (0.007)	
Pre- Use			−0.051 (0.008)
Post-Use			−0.150 (0.012)
Never User		−0.062 (0.005)	−0.061 (0.005)

* All models are adjusted for age, gender, and education.

Annual rate of change in global cognitive functions in Model A is estimated from a mixed-effects model to determine the cognitive trajectory for global cognitive functions over time for two groups: prevalent users to non-prevalent users; Model B uses a mixed-effects model to determine the cognitive trajectory for global cognitive function over time for three study groups: prevalent users, incident users, to never users; Model C is derived from a mixed effect model for three study groups as in Model B but the incident users have a change point defined by the year when a medication with anticholinergic activity was first initiated.

### Medications with anticholinergic activity and change in episodic memory function

As alterations of the cholinergic system have been reported to be associated with changes in episodic memory [25], we also examined whether one of the changes in global cognitive functions associated with initiating a medication with anticholinergic activity was associated with changes in episodic memory. As shown in Table 3, the annual rate of episodic memory change was not different between prevalent users and non-prevalent users. However, in models dividing non-prevalent users into never users and incident users, the incident users had a steeper annual rate of decline in their episodic memory (difference = −0.042 z-score units/year, SE = 0.010, t_7028_ = −4.05, p<0.001). When the episodic memory trajectory of incident users was further delineated into pre-initiation and post-initiation values, post-initiation annual rate of episodic memory decline was 2.7-fold more rapid than never users (difference = −0.098 z-score units/year, SE = 0.016, t_203_ = −6.21, p<0.001) and pre-initiation annual rate of decline did not differ from never users.

**Table 3:** Annual rate of change in episodic memory by user group.

	Annual Rate of Change in Episodic Memory (SE), z-score units		
Model:*	A	B	C
**User Type:**			
Prevalent User	−0.082 (0.015)	−0.080 (0.015)	−0.084 (0.020)
Non-Prevalent User	−0.075 (0.006)		
Incident User		−0.102 (0.009)	
Pre- Use			−0.056 (0.011)
Post-Use			−0.156 (0.014)
Never User		−0.060 (0.007)	−0.058 (0.007)

* All models are the same as described for Table 2 except that episodic memory function replaced global cognitive functions as the outcome.

### Medications with anticholinergic activity, covariates, and change in global cognitive functions

Given our findings that initiation of a medication with anticholinergic activity was associated with a steeper annual rate of decline in global cognitive and episodic memory functions, we wanted to focus on participants initiating such a medication to determine how factors other than using a medication with anticholinergic function (covariates) may have influenced these results. We constructed a mixed-effects model for participants with initiation of a medication with anticholinergic activity to determine the cognitive function trajectory as a function of time in study with a change point placed in the year of initiation of such a medication. In our primary model, we found that only older age at study entry modified the difference in annual rate of global cognitive function decline post-initiation as compared to pre-initiation (parameter estimate for difference in annual rate of change in global cognitive functions at time of medication with anticholinergic activity initiation x age = −0.005, SE = 0.002, p = 0.03). Other baseline covariates (the presence of an apolipoprotein E ε4 allele, the presence of MCI, the number of chronic medical conditions, report of urinary incontinence, the number of depressive symptoms, the level of physical activity, or the presence of basic activities of daily living disability) did not modify the difference in the annual rate of cognitive decline before and after starting a medication with anticholinergic activity (Table S4).

## Discussion

In this cohort of 896 community-dwelling older persons without baseline dementia examined annually for an average of 10 years, initiation of a medication with anticholinergic activity was associated with a 1.8-times steeper decline in global cognitive functions as compared to never users. In addition, after the initiation of a medication with anticholinergic properties, the annual rate of cognitive decline of these participants was 2.9-fold more rapid than prior to initiating a medication with anticholinergic activity.

Identifying modifiable risk factors affecting cognitive decline in older persons is an important public health issue. In the United States, the number of persons over age 65 will double within the next 25 years [26]. Cognitive decline is estimated to affect over 80% of persons over age 70 [1], [2]. The results of this study have important translational consequences and suggest that clinicians caring for older persons need to judiciously consider the initiation of medications with anticholinergic activity.

Prospective longitudinal cohort studies on the relationship between use of medications with anticholinergic activity and cognitive decline are limited. Most [8]–[10], but not all [11], studies of community-dwelling elders without dementia demonstrated that prevalent use of medications with anticholinergic activity was associated with subsequent decline in cognitive function over four to six years. Interpretation of prevalent use studies is limited by not having information regarding cognitive performance before starting medications. Also, the cognitive trajectory of non-prevalent users (i.e., the control group) is a blend of never users and incident users. Therefore, findings among studies can vary depending on the percentage of persons in the control group that become incident users during the course of cognitive follow-up. A novel feature and strength of this study involves being able to model cognitive function over time before and after the first year a medication with anticholinergic activity was started. Also, since follow-up ranged up to 18 years, the long-term effect of initiating a medication with anticholinergic activity on the annual rate of change in cognition was determined. Further emphasizing the risk of using a medication with anticholinergic activity is the finding that prior to initiating a medication with anticholinergic activity, the incident users' rate of cognitive decline was not different than that of never users, whereas after starting a medication the rate of decline became significantly greater.

Our study did find a gradation in the annual rate of cognitive decline with incident users having the greatest decline and never users having the least. However, we did not find a significant difference between the cognitive trajectory of prevalent users and never users in any of our analytic models. One possible explanation is that we did not have enough power to detect a significant difference between prevalent users and never users. An alternative explanation is that the mix of medications with anticholinergic activity in prevalent users had a lower anticholinergic potency than the mix of medications with anticholinergic activity in incident users. One additional possibility for the greater decline may be advanced age, as the incident users were older at time of initiation of a medication with anticholinergic activity than the prevalent users. With aging, there is a dramatic loss of the cholinergic neurons in the basal forebrain [27]. In addition, studies on aging have noted alterations in choline transport, acetylcholine release, nicotinic and muscarinic receptor expression, neurotrophin support, and impaired axonal transport [28]. The basal forebrain cholinergic network is critical in providing both the neocortex and the hippocampus with acetylcholine. Since the prevalent users were younger when placed on medications with anticholinergic activity and had less deterioration of their basal forebrain cholinergic network, they may have been able to up-regulate this cholinergic network. In contrast, because of the age-related decrements in this network in older persons, the initiation of medications with anticholinergic activity may have reduced their brain's capacity to store new information, impairing episodic memory, as well as their ability to retrieve and use previously stored information, inducing a cognitive decline. While medications with anticholinergic activity block acetylcholine, many of these medications are not reported to cross the blood-brain barrier. However, age-related breakdown in the blood-brain barrier may result in easier penetration of these molecules into the brain [28]. Also, reduced systemic clearance of medications due to age-related changes in the liver or kidney [29] may result in higher serum drug concentrations than typically associated with particular medications in younger persons.

Study limitations include the cohort being composed of Catholic clergy, whose level of education and other life experiences differs from the general population. We were not able to examine the effects of total anticholinergic burden using medication dose or serum anticholinergic activity; however, serum activity can be influenced by sampling time. Strengths of our study include the use of a comprehensive neuropsychological battery to assess cognition yearly for an average duration of ten years in a large cohort of participants who were without dementia and were tested both before starting and when using medications with anticholinergic activity. Additional studies are needed to better define if the decline associated with the use of these medications is being induced by the medications' anticholinergic activity, the diseases for which they were being used, or the interaction of these factors. If these medications are playing an important role in cognitive decline, further research is needed to better understand the pathophysiology of cognitive functions decline associated with the use of these medications. Learning how this decline may be prevented in older persons who need to use these medications also will be clinically important.

## Supporting Information

Table S1 Technical Supplement for Model Construction with Change Point for Initiation of a Medication with Anticholinergic Activity.
(DOCX)

Table S2 Values given to time terms at each visit for persons classified by use of medication with anticholinergic activity.
(DOCX)

Table S3 Use of terms to determine average annual rate of change in cognitive function for participant classified by use of a medication with anticholinergic activity.
(DOCX)

Table S4 Effect of baseline covariates on modifying trajectory of global cognitive function in incident users (n = 237) of a medication with anticholinergic activity.
(DOCX)

## References

[1] Barnes DE, Cauley JA, Lui LY, Fink HA, McCulloch C, et al (2007) Women who maintain optimal cognitive function into old age. J Am Geriatr Soc 55: 259–264. 17302664 10.1111/j.1532-5415.2007.01040.x

[2] Yaffe K, Lindquist K, Vittinghoff E, Barnes D, Covinsky KE, et al (2010) The effect of maintaining cognition on risk of disability and death. J Am Geriatr Soc 58: 889–894. 20406308 10.1111/j.1532-5415.2010.02818.x PMC2924918

[3] Hilmer SN, Mager DE, Simonsick EM, Cao Y, Ling SM, et al (2007) A Drug Burden Index to define the functional burden of medications in older people. Arch Intern Med 167: 781–787. 17452540 10.1001/archinte.167.8.781

[4] Lechevallier-Michel N, Molimard M, Dartigues JF, Fagrigoule C, Fourrier-Reglat A (2004) Drugs with anticholinergic properties and cognitive performance in the elderly: results from the PAQUID study. Br J Clin Pharmacol 59: 143–151. 10.1111/j.1365-2125.2004.02232.x PMC1884748 15676035

[5] Merchant RA, Li B, Yap KB, Ng TP (2009) Use of drugs with anticholinergic effects and cognitive impairment in community-living older persons. Age Ageing 38: 105–108. 19008305 10.1093/ageing/afn240

[6] Ancelin ML, Artero S, Portet F, Dupuy AM, Touchon J, et al (2006) Non-degenerative mild cognitive impairment in elderly people and use of anticholinergic drugs: longitudinal cohort study. BMJ 332: 455–459. 16452102 10.1136/bmj.38740.439664.DE PMC1382539

[7] Campbell N, Boustani M, Limbil T, Ott C, Fox C, et al (2009) The cognitive impact of anticholinergics: a clinical review. Clin Interv Aging 4: 225–233. 19554093 10.2147/cia.s5358 PMC2697587

[8] Carriere I, Fourrier-Reglat A, Dartigues JF, Rouaud O, Pasquier F, et al (2009) Drugs with anticholinergic properties, cognitive decline, and dementia in an elderly general population: the 3-City Study. Arch Intern Med 169: 1317–1324. 19636034 10.1001/archinternmed.2009.229 PMC2933398

[9] Boustani MA, Campbell NL, Munger S, Maidment I, Fox GC (2008) Impact of anticholinergics on the aging brain: A review and practical application. Aging Health 4: 311–320.

[10] Fox C, Richardson K, Maidment ID, Savva GM, Matthews FE, et al (2011) Anticholinergic medication use and cognitive impairment in the older population: The Medical Research Council Cognitive Function and Ageing Study. J Am Geriatr Soc 59: 1477–1483. 21707557 10.1111/j.1532-5415.2011.03491.x

[11] Bottigi KA, Salazar JC, Yu L, Caban-Holt AM, Ryan M, et al (2006) Long-term cognitive impact of anticholinergic medications in older adults. Am J Geriatr Psychiatry 14: 980–984. 17068321 10.1097/01.JGP.0000224619.87681.71

[12] Bennett DA, Schneider JA, Arvanitakis Z, Wilson RS (2012) Overview and findings from the Religious Orders Study. Curr Alzheimer Res 9: 628–645. 22471860 10.2174/156720512801322573 PMC3409291

[13] Wilson RS, Evans DA, Bienias JL, Mendes de Leon CF, Schneider JA, et al (2003) Proneness to psychological distress is associated with risk of Alzheimer's disease. Neurology 61: 1479–1485. 14663028 10.1212/01.wnl.0000096167.56734.59

[14] Wilson RS, Mendes de Leon CF, Barnes LL, Schneider JA, et al (2002) Participation in cognitively stimulating activities and risk of incident Alzheimer's disease. JAMA 287: 742–748. 11851541 10.1001/jama.287.6.742

[15] McKhann G, Drachman D, Folstein M, Katzman R, Price D, et al (1984) Clinical diagnosis of Alzheimer's disease: report of the NINCDS-ADRDA Work Group under the auspices of Department of Health and Human Services Task Force on Alzheimer's disease. Neurology 34: 939–944. 6610841 10.1212/wnl.34.7.939

[16] Aggarwal NT, Wilson RS, Beck TL, Bienias JL, Bennett DA (2005) Mild cognitive impairment in different function domains and incident Alzheimer's disease. J Neurol Neurosurg Psychiatry 76: 1479–1484. 16227534 10.1136/jnnp.2004.053561 PMC1739422

[17] Folstein MF, Folstein SE, McHugh PR (1975) ‘Mini-Mental State’: a practical method for grading the cognitive state of patients for the clinician. J Psychiatr Res 12: 189–198. 1202204 10.1016/0022-3956(75)90026-6

[18] Wilson RS, Barnes LL, Krueger KR, Hoganson G, Bienias JL, et al (2005) Early and late life cognitive activity and cognitive systems in old age. J Int Neuropsychol Soc 11: 400–407. 16209420

[19] Hixson J, Vernier DO (1990) Restriction isotyping of human apolipoprotein E by gene amplification and cleavage with Hha I. J Lipid Res 31: 545–548. 2341813

[20] Radloff LS (1977) The CES-D Scale: A self-report depression scale for research in the general population. Appl Psychol Meas 1: 385–401.

[21] McPhillips JB, Pellettera KM, Barrett-Conner E, Wingard DL, Criqui MH (1989) Exercise patterns in a population of older adults. Am J Prev Med 5: 65–72. 2730794

[22] Katz S, Akpom C (1976) A measure of primary sociobiological functions. Int J Health Serv 6: 493–508. 133997 10.2190/UURL-2RYU-WRYD-EY3K

[23] Buchman AS, Shah RC, Leurgans SE, Boyle PA, Wilson RS, et al (2010) Musculoskeletal pain and incident disability in community-dwelling older adults. Arthritis Care Res (Hoboken) 62: 1287–1293. 20853470 10.1002/acr.20200 PMC3445435

[24] Fitzmaurice GM, Laird NM, Ware JH (2011) Applied Longitudinal Analysis, 2^nd^ Edition. Wiley, New York, NY

[25] Hasselmo ME (2006) The role of acetylcholine in learning and memory. Curr Opin Neurobiol 16: 710–715. 17011181 10.1016/j.conb.2006.09.002 PMC2659740

[26] He W, Sengupta M, Velkoff VA, DeBarros KA (2005) U.S. Census Bureau, Current Population Reports, P23-209, 65+ in the United States: U.S. Government Printing Office, Washington, DC, 2005.

[27] McGeer PL, McGeer EG, Suzuki J, Dolman CE, Nagai T (1984) Aging, Alzheimer's disease, and the cholinergic system of the basal forebrain. Neurology 34: 741–745. 6539435 10.1212/wnl.34.6.741

[28] Popescu BO, Toescu EC, Popescu LM, Bajenaru O, Muresanu DF, et al (2009) Blood-brain barrier alterations in ageing and dementia. J Neurol Sci 283: 99–106. 19264328 10.1016/j.jns.2009.02.321

[29] Epstein M (1996) Aging and the kidney. J Am Soc Nephrol 7: 1106–1122. 8866401 10.1681/ASN.V781106

