# Supplementary material for: Cognitive Decline in Older Persons Initiating Anticholinergic Medications
Source: PLoS One. 2013 May 31;8(5):e64111. doi: 10.1371/journal.pone.0064111 (PMC3669362; doi:10.1371/journal.pone.0064111)
Supplement: Table S1 — Technical Supplement for Model Construction with Change Point for Initiation of a Medication with Anticholinergic Activity. (DOCX) [file pone.0064111.s001.docx]

**Table S1. Technical Supplement for Model Construction with Change Point for Initiation of a Medication with Anticholinergic Activity**

We used a mixed effects model^24^ in which the change in cognitive function over time follows a piecewise linear trajectory with the change point defined at the year when initiation of a medication with anticholinergic activity was started. Person-specific trajectory was modeled with random intercept and slopes. The model structure is specified as below,

$$E(y_{ij})=(\theta_{i0}+\gamma_{i0})+(\theta_{i1}+\gamma_{i1})T_{ij}+(\theta_{i2}+\gamma_{i2})(T_{ij}-T'_{i})_{+}$$

Here, $E(y_{ij})$is the expected level of cognitive function for subject *i* at visit *j*. $T_{ij}$ is time (in years) for subject *i* at visit *j*. To differentiate the rate of change in cognition before and after the time of initiation of a medication with anticholinergic activity (denoted by $T'_{i}$), we specify two separate column vectors of time in the model’s design matrix, $T_{ij}$and ($T_{ij}-T'_{i}$)_+_ , Here ($T_{ij}-T'_{i}$)_+_  takes value of zero if $T_{ij}\leq T'_{i}$ and $T_{ij}-T'_{i}$ if $T_{ij}>T'$. $\theta_{i0}+\gamma_{i0}$, $\theta_{i1}+\gamma_{i1}$, and $\theta_{i2}+\gamma_{i2}$ represent separately the subject-specific intercept, the slope before and after the initiation of the medication. The fixed effects,$\mathbf{\theta}=(\theta_{i0},\theta_{i1},\theta_{i2},\theta_{i3})'$, are modeled as linear functions of age, sex, and education, and the random effects $\mathbf{\gamma}=(\gamma_{i0},\gamma_{i1},\gamma_{i2})'$assume a multivariate normal distribution with means 0s and unknown variance covariance structure.

We estimated the slopes from 4 different terms, the first of which was the slope of cognitive function prior to first use of a medication with anticholinergic activity in incident users. The other three terms were adjustments to obtain slopes after initiation or for other users. We compared the differences in the annual rate of cognitive change between various pairs of user groups (prevalent users vs. never users; incident users, pre-use vs. never users; incident users, post-use vs. never users; and incident users, post-use vs. incident users, pre-use).

**Definitions**

Term 1: the slope of cognitive function prior to first use of a medication with anticholinergic activity in incident users

Term 2: the amount by which the slope changes after the initiation of a medication with anticholinergic activity. This second term is negative if decline is steeper after initiation of medication; it is positive if the decline is slower; and it is 0 if there is no change in slope of cognitive function

Term 3: the difference between the slope for prevalent user and the post-initiation slope of incident users

Term 4: the difference between the slopes of never-users and pre-use incident users
