# Supplementary material for: Cognitive Decline in Older Persons Initiating Anticholinergic Medications
Source: PLoS One. 2013 May 31;8(5):e64111. doi: 10.1371/journal.pone.0064111 (PMC3669362; doi:10.1371/journal.pone.0064111)
Supplement: Table S2 — Values given to time terms at each visit for persons classified by use of medication with anticholinergic activity. (DOCX) [file pone.0064111.s002.docx]

**Table S2: Values given to time terms at each visit for persons classified by use of medication with anticholinergic activity**

| Classification of Person by Use of Medication with Anticholinergic Activity Use | Use of Medication with Anticholinergic Activity (Yes/No) | Constructed variable for a given visit | | | |
| --- | --- | --- | --- | --- | --- |
|  |  | Term 1 | Term 2 | Term 3 | Term 4 |
| Never User | No | Time | 0 | 0 | Time |
| Prevalent User | Yes | Time | Time | Time | 0 |
| Incident User | No (Pre Use) | Time | 0 | 0 | 0 |
|  | Yes (Post Use) | Time | (Time since started)_+_ | 0 | 0 |
