# Supplementary material for: Cognitive Decline in Older Persons Initiating Anticholinergic Medications
Source: PLoS One. 2013 May 31;8(5):e64111. doi: 10.1371/journal.pone.0064111 (PMC3669362; doi:10.1371/journal.pone.0064111)
Supplement: Table S3 — Use of terms to determine average annual rate of change in cognitive function for participant classified by use of a medication with anticholinergic activity. (DOCX) [file pone.0064111.s003.docx]

**Table S3: Use of terms to determine average annual rate of change in cognitive function for participant classified by use of a medication with anticholinergic activity**

| Condition of participant | Average annual rate of change in cognitive function |
| --- | --- |
| Never User | Term 1 + Term 4 |
| Prevalent User | Term 1 + Term 2 + Term 3 |
| Incident User: Pre-Use | Term 1 |
| Incident User, Post-Use | Term 1 + Term 2 |
