# Supplementary material for: Cognitive Decline in Older Persons Initiating Anticholinergic Medications
Source: PLoS One. 2013 May 31;8(5):e64111. doi: 10.1371/journal.pone.0064111 (PMC3669362; doi:10.1371/journal.pone.0064111)
Supplement: Table S4 — Effect of baseline covariates on modifying trajectory of global cognitive function in incident users (n = 237) of a medication with anticholinergic activity. (DOCX) [file pone.0064111.s004.docx]

**Table S4. Effect of baseline covariates on modifying trajectory of global cognitive function in incident users (n=237) of a medication with anticholinergic activity.^*^**

| Baseline Covariate | Parameter Estimate for Covariate X Difference in  Post-Use and Pre-Use  Annual Change of Global Cognitive Function (standard error, p-value)^‡^ |
| --- | --- |
| Presence of ApoE ε4 Allele | -0.005 (0.029, 0.8) |
| Mild Cognitive Impairment | 0.019 (0.029, 0.5) |
| Number of Chronic Medical Conditions | -0.010 (0.013, 0.5) |
| Urinary Incontinence | 0.009 (0.025, 0.7) |
| CES-D | 0.011 (0.008, 0.2) |
| Physical Activity | 0.002 (0.002, 0.5) |
| Katz Activities of Daily Living disability | -0.022 (0.043, 0.6) |

* From mixed effects models with terms for annual rate of change in global cognitive function before initiating a medication with anticholinergic activity, the difference in the annual rate of change in global cognitive function post- versus pre-use of such a medication, age, gender, education level, the baseline covariate of interest, and each variable’s interaction with annual rate of change in global cognitive function pre-use of a medication with anticholinergic activity and with the difference in the annual rate of change in global cognitive function post- versus pre-use. Each covariate of interest was added individually into a model adjusted for age, gender, and education. ApoE denotes apolipoprotein E genotype, and CES-D is the score on the Center for Epidemiologic Studies Depression Scale.

^‡^ Represents effect of the covariate of interest on the difference in annual rate of global cognitive function change post- and pre-use for a 75-year old female participant with 18 years of education
